# Supplementary material for: Temperature Dependence of Electrical and Thermal Conduction in Single Silver Nanowire
Source: Sci Rep. 2015 Jun 2;5:10718. doi: 10.1038/srep10718 (PMC4451791; doi:10.1038/srep10718)
Supplement: Supplementary Information [file srep10718-s1.pdf]

Temperature Dependence of Electrical and Thermal Conduction in Single Silver Nanowire

Zhe Cheng, Longju Liu, Shen Xu, Meng Lu, Xinwei Wang*

*Corresponding author. Email: xwang3@iastate.edu, Tel: 515-294-2085, Fax: 515-294-3261

Uncertainty analysis

There are a few factors in the experiment which would affect the accuracy of the measurement results. Here we will have a discussion about them. First, the electrical contact resistance between the silver nanowire and the electrodes is estimated. The Pt pads deposited by EBID are large and good enough to keep good electrical contact. We conducted experiments on silver nanowire without EBID and silver nanowire with silver paste-enhanced contact. In both circumstances, to achieve 1% electrical resistance rise, the applied electrical currents increased after decreasing as the temperature went down. That is because the electrical contact resistance is weakly temperature dependent and the contact resistance dominates the total electrical resistance at low temperatures. The intrinsic electrical resistance needs to rise far more than 1% at low temperatures. That is why large electrical currents are needed at low temperatures. Here the low temperatures means above 25 K because the electrical resistivity becomes weakly temperature dependent when temperature is below 25 K. This would also lead to large applied electrical current to achieve 1% electrical resistance rise. But for the silver nanowire with EBID, the needed electrical currents to achieve 1% resistance rise did not increase at low temperatures. The electrical contact resistance between deposited film and Pt nanowire is also reported negligible in the literature.[1] For the thermal contact resistance after EBID, the Pt-EBID has a contact conductance (h_{con}) of 170.5 MW/(K·m^2) at 293 K.[2] The Pt pad is about 5 μm long for each end and the diameter of the silver nanowire is 227 nm. So the contact area (A_{con}) is 1.78 μm^2 per end and the thermal contact resistance [$1/(A_{con}h_{con})$] between the silver nanowire and Pt pads is 3.3×10^3 K/W per end. The two thermal contact resistances are in parallel so the total thermal contact resistance is 1.65×10^3 K/W. For the silver nanowire, the effective thermal resistance is $\Delta T/q = L/(12\kappa A_c)$. This thermal resistance is defined using the average temperature rise of the

sample and the total heat flux through the sample (the joule heat generated by the sample). Here, L and A_c is the silver nanowire length and cross section area. κ is the thermal conductivity of the silver nanowire. The thermal resistance of the silver nanowire is 2.9×10^5 K/W at room temperature. The thermal contact resistance is very small compared with the thermal resistance of the silver nanowire. So the thermal contact resistance is negligible in this work. The thermal contact resistance is also reported negligible in the literature. [1,2] The length and diameter of the silver nanowire were measured by SEM. The relative errors of the length and diameter measurement are estimated as 1% and 3% respectively. The current error is 0.5% and the voltage error is 0.3%. The relative error of the thermal conductivity and the electrical resistivity are estimated as 7.4% and 4.4% respectively.

Reference

1 Volklein, F., Reith, H., Cornelius, T. W., Rauber, M. & Neumann, R. The experimental investigation of thermal conductivity and the Wiedemann-Franz law for single metallic nanowires. *Nanotechnology* **20**, 32 (2009).

2 Bifano, M. F. P., Park, J., Kaul, P. B., Roy, A. K. & Prakash, V. Effects of heat treatment and contact resistance on the thermal conductivity of individual multiwalled carbon nanotubes using a Wollaston wire thermal probe. *J. Appl. Phys.* **111**, 5 (2012).
